# Brown tumor of the maxillary sinus in a patient with primary hyperparathyroidism: a case report

**DOI:** 10.4076/1752-1947-3-7495

**Published:** 2009-07-06

**Authors:** Efklidis Proimos, Theognosia S Chimona, Dimetrio Tamiolakis, Michalis G Tzanakakis, Chariton E Papadakis

**Affiliations:** 1 ENT Department, Chania General Hospital, Chania, Crete, Greece; 2 Department of Cytopathology, Chania General Hospital, Chania, Crete, Greece

## Abstract

**Introduction:**

Brown tumors are rare focal giant-cell lesions that arise as a direct result of the effect of parathyroid hormone on bone tissue in some patients with hyperparathyroidism. Brown tumors can affect the mandible, maxilla, clavicle, ribs, and pelvic bones. Therefore, diagnosis requires a systemic investigation for lesion differentiation.

**Case presentation:**

We present a 42-year-old Greek woman, with a rare case of brown tumor of the maxillary sinus due to primary hyperparathyroidism. Primary hyperparathyroidism is caused by a solitary adenoma in 80% of cases and by glandular hyperplasia in 20%.

**Conclusions:**

Differential diagnosis is important for the right treatment choice. It should exclude other giant cell lesions that affect the maxillae.

## Introduction

Brown tumors are rare sequelae of hyperparathyroidism. The lesions localize in areas of intense bone resorption, and the bone defect becomes filled with fibroblastic tissue. These tumors have a brown or yellow hue [1]. Brown tumors arise secondary to both primary and secondary hyperparathyroidism, and have been reported to occur in 4.5% of patients with primary hyperparathyroidism and 1.5 to 1.7% of those with secondary disease [1].

Hyperparathyroidism is frequently caused by the development of a parathyroid adenoma and less often by hyperplasia or a carcinoma. Some parathyroid adenomas and hyperplasias are familial (5% of cases), and others are part of multiple type I, IIa, and IIb endocrine neoplasias [2].

In a review of 220 patients, Rosenberg and Guralnick found that 10 patients (4.5%) had a mass involving one or both jaws as their presenting complaint [3]. Brown tumors are more commonly seen in the mandible than in the maxilla [1]. The reported prevalence of brown tumors is 0.1%. The disease can manifest at any age, but it is more common among persons older than 50 years, and is three times more common in women than in men [2].

Most patients with hyperparathyroidism are asymptomatic. Hypercalcemia is often discovered incidentally during routine laboratory testing; hypophosphatemia and increased alkaline phosphatase levels in blood may also be seen [2]. Any of the skeletal bones may be affected, including the cranio-maxillofacial ones. Brown tumors may be the first clinical sign of hyperparathyroidism. Brown tumors are a localized form of fibrous-cystic osteitis found in the presence of hyperparathyroidism [1,2]. Histologically, brown tumors are made up of mononuclear stromal cells mixed with multinucleated giant cells, among which recent hemorrhagic infiltrates and hemosiderin deposits (hence the brown color) are often found [3]. Whenever a round, radiolucent, and bone-expanding lesion in the facial bones of a patient with hyperparathyroidism is presented, a brown tumor must be considered to be the most likely diagnosis [1,2]. However, when the same type of lesion is found in patients without hyperparathyroidism, a more complex differential diagnosis procedure must be followed [2,3].

## Case presentation

**Figure 1:**
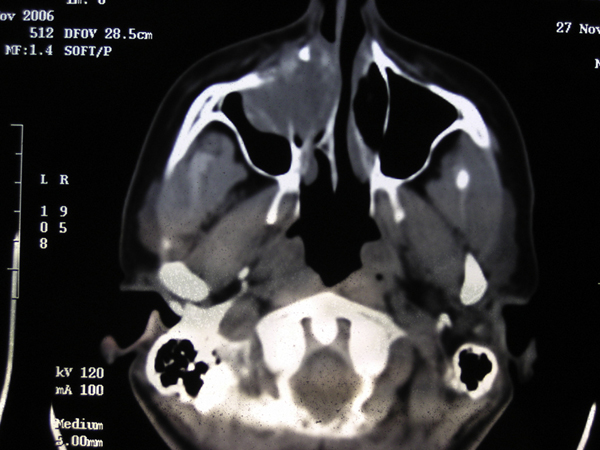
**Axial computed tomography of the paranasal sinuses showing a round osteolytic lesion of the right maxillary sinus**.

**Figure 2:**
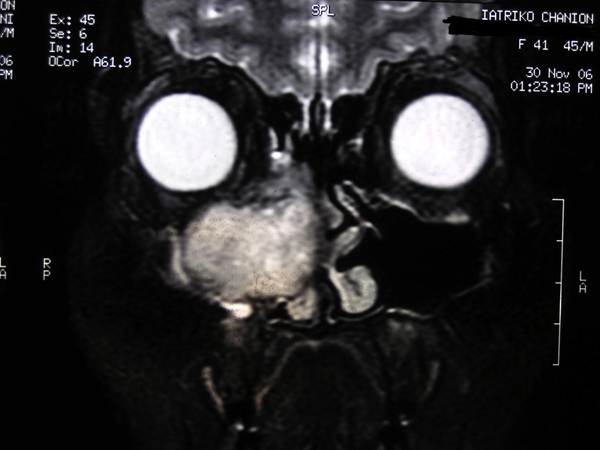
**Coronal magnetic resonance imaging of the paranasal sinuses showing a round, bone-expanding lesion in the anterior part of the right maxillary sinus with invasion of the adjacent floor of the orbit, anterior ethmoids and nasal cavity**.

A 42-year-old Greek woman was referred to the Department of Otolaryngology of Chania General Hospital, complaining of facial pain and deformity, and a radiographic finding of a round, radiolucent, and osteolytic, bone-expanding lesion in the anterior part of the right maxillary sinus with invasion of the adjacent floor of the orbit, anterior ethmoids and nasal cavity (Figures 1 and 2). There was no family history of peptic ulcer, parathyroid disease, or any other endocrinopathy. There was no history of increased thirst, urinary frequency, constipation, weight loss, urolithiasis, fracture, peptic-ulcer, use of vitamin or calcium supplements, Paget's disease, exposure to ionizing radiation or industrial toxins, or use of tobacco or alcohol.

On physical examination, the patient appeared well. Her head and neck were normal; no cervical masses were palpated. Her lungs, heart, breasts, and abdomen were normal, as was her urine. Hematocrit was 39.8%; white-cell count was 6800/µL, with a normal differential count; platelet count, prothrombin time, and partial-thromboplastin time were normal. Rigid nasal endoscopy, with a 0^0^ endoscope, revealed medialization of the right lateral nasal wall with no signs of mucosal alteration.

**Figure 3:**
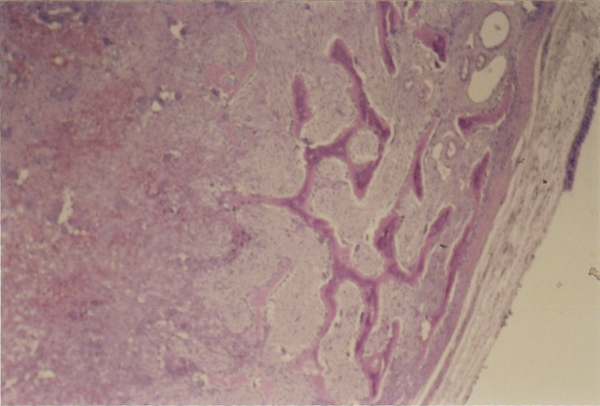
**Brown tumor**. Bone lesion with multinucleated giant cells, mononuclear stromal cells, osteoblastic and osteoclastic areas and deposits of hemosiderin. Hematoxylin and eosin stain, ×100.

Under general anesthesia, an excision biopsy was performed using the Caldwell-Luc approach. The mass had a submucosal origin from the floor of the orbit, infiltrating the lateral nasal wall, with a diameter of approximately 2 cm. The mucosa over the mass was intact and after its excision, erosion of the floor of the orbit was detected with prolapsed periorbital fat. The opening was repaired with an underlay application of dura tissue which was immobilized with tissue glue. Histological sections showed a lesion with regions of discrete bone resorption with osteoclast activity, areas with newly formed bone tissue, and a loose fibrillar matrix with deposits of hemosiderin (Figure 3). Multinucleated giant cells were also found in the lesion (Figure 3). A giant cell lesion was diagnosed, with a brown tumor among the possible causes.

Blood tests after histology demonstrated elevated calcium (13.3 mg/dL) and parathyroid hormone (PTH) concentrations (920 pg/mL). Phosphorus was 1.9 mg/dL, alkaline phosphatase was 227 U/L (normal range 30 to 100 U/L); the urea nitrogen, creatinine, glucose, bilirubin, uric acid, protein, albumin, globulin, serum aspartate aminotransferase, and thyroid-stimulating hormone levels were all normal. Sodium was 139 mmol, and potassium was 5.5 mmol. Carcinoembryonic antigen was less than 1 µg/L, and serum immunoelectrophoresis was normal. The concentrations of IgG, IgA, and IgM were normal. This suggested the diagnosis of primary hyperparathyroidism initially manifesting as a brown tumor of the maxilla. Postoperative explorations with magnetic resonance imaging (MRI) scans and gadolinium infusion confirmed the existence of an underlying parathyroid adenoma in the upper left parathyroid gland as the cause of the condition. The patient was referred to the outpatient clinic of the Department of Endocrinology, at the University Hospital of Crete, for examination of her parathyroid function.

## Discussion

Various anatomopathological entities, both benign and malignant, can appear as bone-expanding or lytic lesions in the facial bones [4]. In the case of a lytic region of the jaw bones, the most likely diagnoses would include: odontogenic cysts and tumors (radicular cyst, lateral periodontal cyst, and ameloblastoma), infectious diseases (bone abscess, localized osteomyelitis), metabolic bone disease hyperparathyroidism, metastasis from a known or an unknown primary site (lung, breast, kidney, prostate), primary bone tumors and cysts (simple bone cyst, eosinophilic granuloma, giant cell lesions, odontogenic keratocyst, myxoma and odontogenic fibroma).

Bone-expanding giant cell lesions that can arise in the jaw bones include giant cell tumor, giant cell reparative granuloma, cherubism and brown tumor. As it is difficult to distinguish brown tumors from other giant cell lesions on the basis of histology or radiology examination, the clinical diagnosis is made based in relation to hyperparathyroidism [4]-[6]. In brown tumors, however, there is a combination of osteoblastic and osteoclastic activity. Brown tumors are mainly the result of secondary hyperparathyroidism in patients with renal insufficiency, but they have also been described as a rare manifestation of calcium malabsorption and some forms of osteomalacia [4]-[6]. Brown tumors as a manifestation of primary hyperparathyroidism are extremely rare. In such cases, the primary hyperparathyroidism usually results from the overproduction of parathyroid hormone by a parathyroid tumor [7]-[9]. In our patient, the histological findings were indicative of a giant cell bone expanding lesion with brown tumor being the most probable diagnosis. Blood tests confirmed primary hyperparathyroidism. The treatment of hyperparathyroidism is the first step in the management of brown tumors. Regression and healing of the lesions are expected after the correction of hyperparathyroidism, but several cases have been reported of brown tumors that grew after parathyroidectomy or normalization of hyperparathyroidism levels [10]. In these cases, brown tumor resection should be the treatment of choice.

The incidence of bone lesions in patients with hyperparathyroidism has fallen from 80% to a current 15%, a reduction that is attributed to better hypocalcemia monitoring in asymptomatic patients, and to the wider use of biochemical analyses [11]. In spite of this, however, Dilip *et al*. [12] reported a series of 40 cases that all had generalized bone involvement. Within the osseous manifestations of hyperparathyroidism are brown tumors that appear in approximately 10% of cases and in advanced stages of the disease. These may appear in any part of the skeleton, most commonly seen on the ribs, clavicle and pelvis. Mandibular involvement has been noted in 4.5% of patients. As a result of the direct effect of PTH on bones, a conversion of the osteogenic potential of the cells occurs changing them from osteoblasts to osteoclasts, with bone resorption predominating over the formation of new bone tissue. As a result of intraosseous bleeding and tissue degeneration, cysts may develop; groups of hemosiderin-loaded macrophages, giant cells and fibroblast fill these cystic lesions. Vascularization hemorrhages and hemosiderin deposits give rise to the characteristic color of the lesions and the term brown tumors.

It is important to point out that brown tumors are non-neoplastic lesions that are very similar to giant cell tumors, but in the context of hyperparathyroidism, they are considered reparative granulomas and they do not have the malignant or neoplastic potential of real giant cell lesions. The symptoms caused by these lesions depend on their size and location. In the maxilla, they can cause pain or deformity, as in our patient. In other cases, the lesions were asymptomatic and the diagnosis occurred accidentally as a result of a radiological examination. When a tumor of the maxilla has been diagnosed as a giant cell tumor, hyperparathyroidism should be ruled out to exclude the possibility that it is a brown tumor. Other differential diagnoses include the reparative granuloma of giant cells, giant cell tumors, and fibrous dysplasia. A definitive diagnosis is only possible on comparison of the clinical, radiological and biochemical analyses. Brown tumors exhibit no pathognomonic histologic changes. Examination will reveal a dense fibroblastic stroma, areas of cystic degeneration, osteoid, microfractures, hemorrhage, macrophages with hemosiderin, and multinucleated osteoclastic giant cells. Similar changes may occur in fibrous dysplasia, true giant-cell tumors, and reparative granulomas [13].

Differentiating between a brown tumor and other giant-cell tumors may be very difficult, even with histology. Fibrous dysplasia affects the bones of the face, and it is most common among young women. Histology reveals trabecular bone with a stroma rich in fibrous tissue and multinucleated giant cells that are visible in areas of hemorrhage secondary to focal degeneration [13]. True giant-cell tumors are more infiltrative than brown tumors. Histological analysis reveals giant cells around a fibrous stroma and some degree of cellular atypia [13]. Reparative granulomas are localized tumors detected in young patients. They primarily involve the mandible. Their cause is still unknown, but some investigators believe that they are a result of trauma [13]. A reparative granuloma can be differentiated from a brown tumor by the absence of hyperparathyroidism. Histologically, they contain giant cells, but their stroma is less dense and more vascularized [13]. Therefore, patients with giant-cell tumors should be investigated for the presence of hyperparathyroidism and hypercalcemia in order to differentiate these granulomas from brown tumors.

There is agreement as to the treatment of choice for primary hyperparathyroidism being parathyroidectomy; however, opinions are divided as to the treatment of bone lesions. Authors such as Scott *et al.*[14] believe that bone lesions reappear spontaneously following removal of the diseased parathyroid gland; others such as Martinez-Gavidia *et al.*[10] recommend initial treatment with systemic corticosteroids in order to reduce the tumor size, followed by surgical removal of the residual lesion. In the case of large destructive cysts, the amount of tissue damaged may be so great that there are few possibilities of remodeling once normocalcemia has been achieved. In these situations, or in cases where the lesions continue for more than 6 months, or there is disruption of the function of the affected organ, or growth despite adequate metabolic control, Yamazaki *et al.*[15] recommend curettage and enucleation.

## Conclusions

Due to recent improvements in analytical techniques, the diagnosis of hyperparathyroidism usually occurs when the disease is in an asymptomatic phase, and the incidence of patients with advanced bone lesions is rare. The treatment of choice for bone lesions is a parathyroidectomy; however, in the case of larger lesions, or those that persistently grow in spite of treatment, or those lesions causing incapacity, curettage and associated enucleation should be conducted.

## Consent

Written informed consent was obtained from the patient for publication of this case report and any accompanying images. A copy of the written consent is available for review by the Editor-in-Chief of this journal.

## Competing interests

The authors declare that they have no competing interests.

## Authors' contributions

All authors provided an equal intellectual contribution to this manuscript. PE and CTH analyzed and interpreted the patient data regarding the tumor. DT performed the histological examination of the tumor. The clinical notes were reviewed by CEP. All authors read and approved the final manuscript.
